# Association Between Total Genotype Score and Muscle Injuries in Top-Level Football Players: a Pilot Study

**DOI:** 10.1186/s40798-024-00682-z

**Published:** 2024-03-07

**Authors:** Myosotis Massidda, Laura Flore, Paolo Cugia, Francesco Piras, Marco Scorcu, Naoki Kikuchi, Pawel Cięszczyk, Agnieszka Maciejewska-Skrendo, Filippo Tocco, Carla Maria Calò

**Affiliations:** 1 Department of Medical Sciences and Public Health, University of Cagliari, Cagliari, Italy; 2 Italian Federation of Sports Medicine, Rome, Italy; 3 Department of Life and Environmental Sciences, University of Cagliari, Cagliari, Italy; 4 Cagliari Calcio SpA, Cagliari, Italy; 5 Nippon Sport Science University (NSSU), Tokyo, Japan; 6 Department of Physical Education, University of Physical Education and Sport, Gdańsk, Poland; 7 Institute of Physical Culture Sciences, University of Szczecin, 71-065 Szczecin, Poland

**Keywords:** TGS, Muscle damage, Soccer, Gene

## Abstract

**Background:**

Recently, genetic predisposition to injury has become a popular area of research and the association between a few single nucleotide polymorphisms (SNPs) and the susceptibility to develop musculoskeletal injuries has been shown. This pilot study aimed to investigate the combined effect of common gene polymorphisms previously associated with muscle injuries in Italian soccer players.

**Results:**

A total of 64 Italian male top football players (age 23.1 ± 5.5 years; stature 180.2 ± 7.4 cm; weight 73.0 ± 7.9 kg) were genotyped for four gene polymorphisms [*ACE* I/D (rs4341), *ACTN3* c.1729C > T (rs1815739), *COL5A1* C > T (rs2722) and *MCT1* c.1470A > T (rs1049434)].

Muscle injuries were gathered for 10 years (2009–2019). Buccal swabs were used to obtain genomic DNA, and the PCR method was used to genotype the samples. The combined influence of the four polymorphisms studied was calculated using a total genotype score (TGS: from 0 to 100 arbitrary units; a.u.). A genotype score (GS) of 2 was assigned to the “protective” genotype for injuries, a GS of 1 was assigned to the heterozygous genotype while a GS of 0 was assigned to the “worst” genotype.

The distribution of genotype frequencies in the *ACE* I/D (rs4341), *ACTN3* c.1729C > T (rs1815739) and *MCT1* c.1470A > T (rs1049434) polymorphisms was different between non-injured and injured football players (*p* = 0.001; *p* = 0.016 and *p* = 0.005, respectively). The incidence of muscle injuries was significantly different among the *ACE* I/D (rs4341), *ACTN3* c.1729C > T (rs1815739) and *COL5A1* C > T (rs2722) genotype groups, showing a lower incidence of injuries in the “protective” genotype than “worse” genotype (*ACE*, *p* < 0.001; *ACTN3*, *p* = 0.005) or intermediate genotype (*COL5A1*, *p* = 0.029).

The mean TGS in non-injured football players (63.7 ± 13.0 a.u.) was different from that of injured football players (42.5 ± 12.5 a.u., *p* < 0.001). There was a TGS cut-off point (56.2 a.u.) to discriminate non-injured from injured football players. Players with a TGS beyond this cut-off had an odds ratio of 3.5 (95%CI 1.8–6.8; *p* < 0.001) to suffer an injury when compared with players with lower TGS.

**Conclusions:**

These preliminary data suggest that carrying a high number of "protective" gene variants could influence an individual's susceptibility to developing muscle injuries in football. Adapting the training load parameters to the athletes’ genetic profile represents today the new frontier of the methodology of training.

## Key Points

- The* ACE* I/D (rs4341),* ACTN3* c.1729C>T (rs1815739),* COL5A1* C>T (rs2722) and* MCT1* c.1470A>T (rs1049434) polymorphisms (and their interaction) are associated with muscle injury in football players.
- The * ACE* D allele, the * ACTN3* C allele, the * COL5A1* C allele, and the * MCT1* T allele seem to protect football players from developing muscle injuries.
- The Total Genotype Score was associated with the incidence of muscle injuries in Italian top-level footballers, and this result needs replication in a larger independent cohort.

## Background

Muscle injuries are one of the most frequent traumatic events during sports, particularly in football [1]. They are difficult to define and characterize due to their heterogeneity and their complex etiology [2] regulated by environmental and genetic factors. Coaches, physiologists, and the medical community are interested in identifying the variables that predispose athletes to more or lesser risk of muscle damage in order to identify the risk factors and implement various recovery tactics and specialized training approaches [3]. Genetics play a vital role in sports performance, and it is increasingly recognized as a significant risk factor for injury. The expression of certain genes affects muscles, tendons, and ligaments and consequently sports performance [4], and various football athletic abilities and skills such as jumping, sprinting, repeated sprint ability, and training response [5, 6]. Recently, we and other researchers have demonstrated an association between several single nucleotide polymorphisms and musculoskeletal injuries in professional football players, and the most studied genetic variants in this context include *ACE* I/D (rs4341) [7–10], *ACTN3* c.1729C > T (rs1815739) [8–10], *COL5A1* C > T (rs2722) [11, 12], and *MCT1* c.1470A > T (rs1049434) [13].

The *ACE* I/D (rs4341) gene variant, which encodes angiotensin-converting enzyme (ACE) in human skeletal muscle, is associated with ACE activity, and it has been the first gene to be examined in relation to human sports performance [14]. The *ACE* I/D (rs4341) polymorphism is associated with several exercise-related phenotypes, including muscle strength [15], muscle metabolism [16], muscle volume [17], cardiac growth response to exercise [14], skeletal muscle fiber distribution and capillarization [18] and resistance to fatigue in response to physical training [19]. Moreover, individuals who have DD or ID genotypes are more at risk of developing hypertension in adulthood than those who have genotype II [20].

In addition, different concentrations of circulating creatine kinase (CK), a marker of exercise-induced muscle damage (EIMD), were also observed in different *ACE* I/D genotypes after eccentric exercise [21]. Specifically, EIMD individuals with one or two copies of the D allele showed lower elevations and peak CK values compared to individuals with the II genotype. Furthermore, the D-allele was discovered to be connected to a reduced CK response after triathlon [22] and marathon [23] races. These results assert that the *ACE* D allele is connected with reduced susceptibility to muscle damage and support the data from Italian and Japanese football players that showed an association between the D allele and a lower incidence of muscle injuries [7]. However, the association of *ACE* I/D (rs4341) with exercise-related phenotypes and physical performance is controversial, with several studies showing no association [24–28].

The *ACTN3* c.1729C > T (rs1815739) gene variant encodes the sarcomeric protein α-actinin-3, a major component of the Z-line in the muscle that anchors the actin-thin filaments. At amino acid, position 577, a frequent null polymorphism in this gene converts an arginine (C) residue into an early stop codon (T). The *ACTN3* c.1729C > T polymorphism is associated with sprint/power performance, and sport specificity [29], with muscle function and muscle strength [30]. In addition, regarding football, the *ACTN3* c.1729C > T gene variant has been associated with a position on the field [31], athlete status [32], career progression [33], as well as the incidence and severity of muscle injuries [34], recovery times [9], and with increased susceptibility to eccentric muscle damage [35].

The rs12722 C > T polymorphism in the 3'-UTR non-coding region of the COL5A1 gene, which encodes the α1 (V) chain of type V collagen, causes a 50% reduction in type V collagen, which results in poorly structured fibrils, decreased tensile strength, and stiffness of connective tissues [36]. The *COL5A1* C > T (rs2722) polymorphism has been associated with anterior cruciate ligament rupture [36], Achilles tendon pathology [37], exercise-related muscle spasms, and muscle injuries in football players [11].

The c.1470A > T (rs1049434) polymorphism in the *MCT1* gene, which causes aspartic acid to replace glutamic acid (E490D), was discovered [38]. The T allele (490-Asp) of the *MCT1* c.1470A > T polymorphism has been associated with a 35–40% reduction in erythrocyte lactate transport rate [38], post-exercise blood lactate concentration, sprint/power performance [39], body composition [40], climbing status [41], and with a lower incidence of muscle injuries in elite football players [13].

Varillas-Delgado et al. [42] recently demonstrated that the genetic distribution in professional football players is different to the non-athlete population, with a “favorability” of the polygenic profile in muscle injuries to professional athlete status in elite endurance athletes and professional football players.

De Almeida et al. [43] and Maestro et al. [10] recently examined the combined impact of some polymorphisms  (*AMPD1* (rs17602729), *ACE* (rs4646994), *ACTN3* (rs1815739), *CKM* (rs8111989) and *MLCK* (rs2849757 and rs2700352) on muscle injuries in professional soccer players and concluded that the likelihood of injuries may be influenced by a polygenic profile of genes involved in muscle performance.

This pilot study aimed to investigate, for the first time, the combined effect of four common gene polymorphisms previously reported to be associated with muscle injuries in elite Italian football players from different levels of competition [7, 11, 13, 34]. We hypothesized that the four polymorphisms included in the analysis and their combined effect could significantly influence muscle injuries in football.

The knowledge derived from the present investigation will add to the literature on the field of the genetic variations associated with a player’s predisposition to muscle injury.

## Methods

### Participants

This longitudinal cohort study analyzed 64 male professional football players (age 23.1 ± 5.5 years; height 179.3 ± 7.3 cm; weight 73.0 ± 7.9 kg) in one Football Club from Serie A in Italy, whose characteristics are reported in Table 2. All the players involved in the study competed in the Official National Football Championship (Serie A). Thirty-one of them were of international level, while the rest of them (*n* = 33) were of National level. The athletes trained for 8 weeks (28 ± 5 h/week) in the preseason period and 38 weeks during the competitive season (10 ± 2 h/week). The inclusion criteria for participants were determined as follows: (a) Football players of Italian Caucasian origin for ≥ 3 descent; (b) with a contract with the first team of the football club; (c) who participated in training and matches throughout the seasons at the same football club; and (d) performed regular exercise training of > 1 h per day, > 5 days per week for the prior 6 months. The exclusion criteria for participants were determined as follows: (a) Football players of Italian Caucasian origin for ≤ 3 descent; and (b) professional female soccer players. The study methodology was authorized by the local ethical committee at the University of Cagliari and complied with the Declaration of Helsinki for Human Research of 1974 (latest revision in 2000). All participants provided their explicit written consent.

### Injury Data Collection

The study's design followed the guidelines provided in the consensus paper and by UEFA [44] for terminology and methods for gathering data in research addressing football injuries. Any musculoskeletal condition that arose during training and kept a player from training or match play for at least one day following the day of commencement was considered an indirect muscle injury. All the football players who suffered direct muscle injuries (contusion and laceration) were included in the analyses, but the direct injuries were not considered as injuries. The injury incidence was determined as follows: (number of muscle injuries/training exposure hours + 1000) × 1000 [45]. "Training exposure" refers to any team-based or individual physical activity done under the direction or supervision of the team's coaches and fitness professionals and created with the intention of maintaining or improving players' football skills or physical condition. Matches competitions were considered as training exposure.

The severity of injuries was gauged by the number of days lost from training and competition. We have defined the injury severity based on the maximum number of days of absence after an injury that has been recorded for that player. Moreover, we used the same guidelines [44] to differentiate the severity of injuries, classifying them into minor (4–7 days of absence), moderate (8–28 days of absence) and major (more than 28 days of absence). Data were gathered for ten years in a row (2009–2019), and injuries were followed up on for 1 to 6 years. For example, the injury data for players traded away during the season were only included for the time they were on the roster. Players with current injuries were not disqualified from the study, but their current injuries were not taken into consideration. The team's medical personnel conducted a clinical evaluation before registering a muscle injury. During the preseason and regular season, the team's medical staff (physicians and coaches) recorded time lost due to injuries every week using a standardized injury report form (FIFA F-Marc—http://www.f-marc.com/). Using ultrasonic and magnetic resonance imaging scans, the injuries were grouped morphologically. The study's classification system for muscle injuries was developed by Muller-Wohlfarth [2] and was based on a consensus statement for sports injuries.

### DNA Analyses

Each participant's buccal swab was taken and put in a tube with 1 ml of ethanol. Genomic DNA was extracted using a buccal swab by the manufacturer's instructions provided with a commercially available kit (Qiagen, Hilden, Germany). The concentration of the isolated DNA was measured using the fluorometric method (Qubit by Invitrogen, Waltham, MA, USA). The recovered DNA typically had a concentration of 20 mg/mL, which is adequate for PCR. The process of genotyping has been discussed elsewhere [7, 11, 13, 34].

### Polygenic Profile for Muscle Injuries

We used Williams & Folland's method [46] to calculate the combined influence of all four polymorphisms that were under investigation. We began by assigning a score to each genotype within each polymorphism (Table 1). We assigned a genotype score (GS) of 2 to the ‘low risk of injury’ genotype, whereas a GS of 0 was assigned to the ‘high risk of injury’ genotype. Second, we summed the GS of every single genotype (GS_ACE_ + GS_ACTN3_ + GS_COL5A1_ + GS_MCT1_). Finally, the total genotype score (TGS), which was converted to a scale of 0–100 arbitrary unit (a.u.) for easy understanding, was as follows:

$${\text{TGS}} = \, \left( {{1}00/{8}} \right){\text{ x }}({\text{GS}}_{{{\text{ACE}}}} + {\text{GS}}_{{{\text{ACTN}}3}} + {\text{GS}}_{{{\text{COL5A1}}}} + {\text{GS}}_{{{\text{MCT1}}}} )$$

where 8 is the result of multiplying 4 (number of studied polymorphisms) by 2, which is the score given to the ‘low risk’ (protective) genotype for injury. A TGS of 100 represents a ‘low risk’ (protective) polygenic profile for developing musculoskeletal injuries—that is, all GS are 2. In contrast, a TGS of 0 represents the ‘high risk’ (worst) polygenic profile for developing musculoskeletal injuries—that is, all GS are 0.

**Table 1:** Genotype distribution in Injured and Non-injured football players

Symbol	Polymorphism	dbSNP	Genotype score	Injured *N* (%)	Non-Injured N (%)	*p* value
*ACE*	I/D	rs4341	2 = DD	8 (22.8)	19 (65.5)	
			1 = ID	23 (65.7)	9 (31.0)	**0.001**
			0 = II	4 (11.4)	1 (3.5)	
*ACTN3*	rs1815739	c.1747C > T	2 = CC	9 (25.7)	13 (44.8)	
			1 = CT	19 (54.2)	16 (55.1)	**0.016**
			0 = TT	7 (20.0)	0 (0.00)	
*COL5A1*	rs12722	C > T	2 = CC	5 (14.2)	7 (24.1)	
			1 = TC	15 (42.8)	16 (55.1)	0.079
			0 = TT	15 (42.8)	6 (20.6)	
*MCT1*	rs1049434	c.1470A > T	2 = TT	2 (5.70)	7 (29.1)	
			1 = AT	14 (40.0)	15 (51.7)	**0.005**
			0 = AA	19 (54.2)	7 (24.1)	

Bold emphasis: *p* < 0.05

### Statistical Analyses

Statistical analysis was performed using the Statistical Package for the Social Sciences (SPSS), v.21.0 for Windows (IBM Corp. Released 2012. IBM SPSS Statistics for Windows, Version 21.0. Armonk, NY: IBM Corp. United States) and with Genepop (Version 4.0.3). Fisher’s method (*X*^2^) was used to determine the Hardy–Weinberg equilibrium for each polymorphism and the genotype and allele frequency distributions in injured and non-injured groups. For the other variables presented as frequency (i.e., position on the field), the differences in distribution were identified with crosstabs and Pearson Chi-Square (*X*^2^) test of independence. The normality of each variable was initially tested with the Kolmogorov–Smirnov tests, and parametric/nonparametric statistics were performed for normally/non-normally distributed variables, respectively. For the continuous variables, group comparisons (i.e., genotypes under the co-dominant model and injured vs non-injured) were performed using a one-way analysis of variance (ANOVA) or Kruskal–Wallis or Mann–Whitney tests. If the comparison result produced a *p* value < 0.05, a post hoc test was used to find out which genotype group means differ from one another. Significance values have been adjusted by the Bonferroni correction for multiple tests. The ability of TGS to correctly distinguish injuries (0 = no, 1 = yes) and severity > 28 days (0 = no, 1 = yes) was assessed using a receiver operating characteristic (ROC) curve [47]. Thus, the area under the ROC curve (AUC) was calculated with confidence intervals of 95% (95%CI). Linear regression has been used to analyze the relationship between TGS and the incidence and severity of muscle injuries. The significance level was set at *P* < 0.05.

## Results

The *ACE* I/D, *ACTN3* c.1729C > T, *COL5A1* C > T and *MCT1* c.1470A > T genotype frequencies (Table 1) did not deviate from Hardy–Weinberg equilibrium (*p* > 0.05).

Table 2 shows the characteristics of the participants. No significant differences were observed in age, stature, weight, training exposure, seasons played, and playing position between the injured and non-injured groups (*p* > 0.05).

**Table 2:** Characteristics of the study participants

	Injured	Non-injured	*p* value
Players *N*, (%) ▲	35 (54.6)	28 (45.4)	
Age, *years*	23.2 ± 5.4	23.0 ± 5.7	0.71
Stature, *cm*	180.2 ± 7.4	178.7 ± 7.3	0.37
Weight, *kg*	74.4 ± 8.4	71.3 ± 7.2	0.14
Training exposure, *h*	1522 ± 482	1133 ± 583	0.47
Seasons played	2.6 ± 1.4	2.1 ± 0.8	0.31
Players position *N*, (%) ■			0.97
Goalkeepers	4 (11.4)	4 (14.2)	
Defenders	10 (28.5)	7 (25.0)	
Midfielders	11 (31.4)	10 (35.7)	
Forwards	10 (28.5)	8 (28.5)	

Values are presented as the mean ± SD unless noted otherwise

▲ *N* = number of players in injured and non-injured groups

▲ (%) = percentage of players in injured and non-injured groups

■ *N* = number of players for each position in injured and non-injured groups

■ (%) = percentage of players for each position in injured and non-injured groups

The mean incidence of injury was 1.3 ± 2.3 muscle injuries per 1000 h of exposure. The average length of time lost due to indirect muscle injury was 58.1 ± 58.1 days. Considering all the injuries, 17.1% were classified as minor, 22.8% as moderate, and 60% as severe.

A significant association was observed between all four SNPs and muscle injuries. The *ACE* I/D genotype frequency was significantly different between the injured and non-injured groups (*X*^*2*^ 13.7, *df* = *2*, *p* = 0.001, Table 1). Moreover, the incidence of muscle injuries was significantly different among genotypes, showing a lower incidence of injuries in the “optimal” genotype (DD = 0.86) than intermediate genotype (ID = 1.36) and “worse” genotype (II = 4.20) (*p* < 0.001). No significant differences in the severity of injuries among genotypes were observed (DD = 73.1, ID = 52.7, II = 59.2, *p* = 0.351).

The *ACTN3* c.1729C > T genotype frequency was significantly different between the injured and non-injured groups (*X*^*2*^ 8.15, *df* = *2*, *p* = 0.016, Table 1). Moreover, the incidence of muscle injuries was significantly different among genotypes, showing a lower incidence of injuries in the “optimal” genotype (CC = 0.5) than “worse” genotype (TT = 3.3) and intermediate genotype (CT = 1.5) (*p* = 0.005). However, after Bonferroni correction, the incidence of muscle injuries was significantly different only between CC vs TT genotype (*p* = 0.005). No significant differences in the severity of injuries among genotypes were observed (CC = 32.8, CT = 73.0, TT = 50.2, *p* = 0.384).

The *COL5A1* C/T genotype frequency was not significantly different between the injured and non-injured groups (*X*^*2*^ 5.07, *df* = *2*, *p* = 0.079, Table 1). However, the incidence of muscle injuries was significantly different among genotypes, showing a higher incidence of injuries in the “worst” genotype (TT = 2.35) than in the intermediate genotype (TC = 0.68) (*p* = 0.029). No significant differences in the severity of injuries among genotypes were observed (CC = 56.6, TC = 44.1, TT = 72.6, *p* = 0.234).

The *MCT1* c.1470A > T genotype frequency was significantly different between the injured and non-injured groups (*X*_*2*_ 10.40, *df* = *2*, *p* = 0.005, Table 1). Moreover, the incidence of muscle injuries was significantly different between genotypes, showing a lower incidence of injuries in the “optimal” genotype (TT = 0.78) than in the “worse” genotype (AA = 2.22) (*p* = 0.031). However, after Bonferroni correction, the incidence of muscle injuries was not significantly different (p = 0.092). No significant differences in the severity of injuries among genotypes were observed (AA = 63.0, AT = 47.9, TT = 83.5, *p* = 0.505).

### Total Genotype Score and Muscle Injuries

**Fig. 1:**
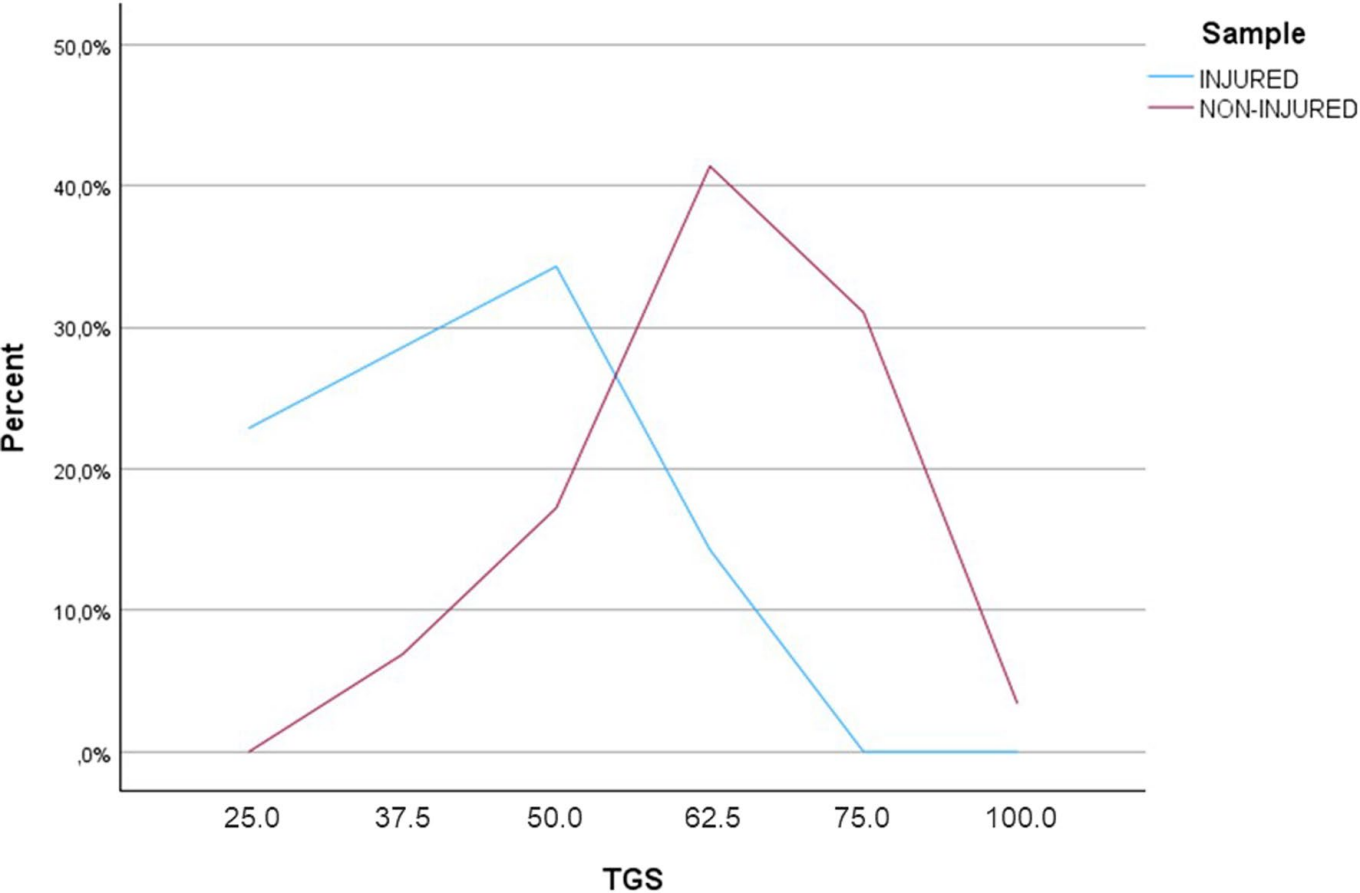
TGS distribution in injured and non-injured football players

When adding the genotype scores of all polymorphisms, the mean value of the TGS in non-injured players was 63.7 ± 13.0 a.u., statistical kurtosis: 1.1, while the mean TGS value for the group of injured players was 42.5 ± 12.5 a.u., statistical kurtosis: -1.0. The distributions of TGS frequencies of the injured and non-injured football players are represented in Fig. 1. The TGS distribution in injured football players was shifted to the right with respect to non-injured football players. The TGS values between non-injured and injured professional football players were significant different (*p* < 0.001).

**Fig. 2:**
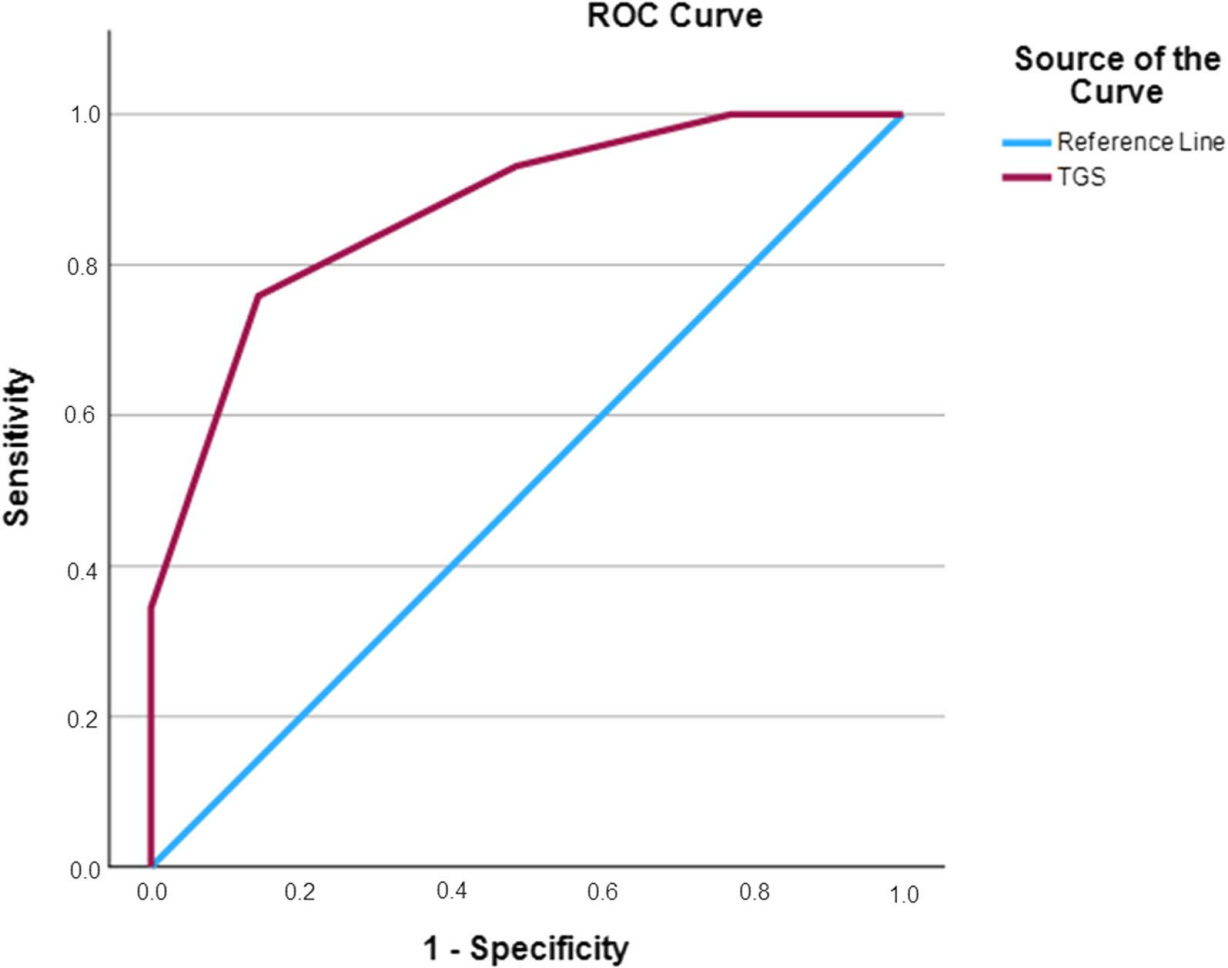
Receiver operating characteristic curve (ROC) summarizing the ability of the total genotype score (TGS) to distinguish potential non-injured players from injured players

**Fig. 3:**
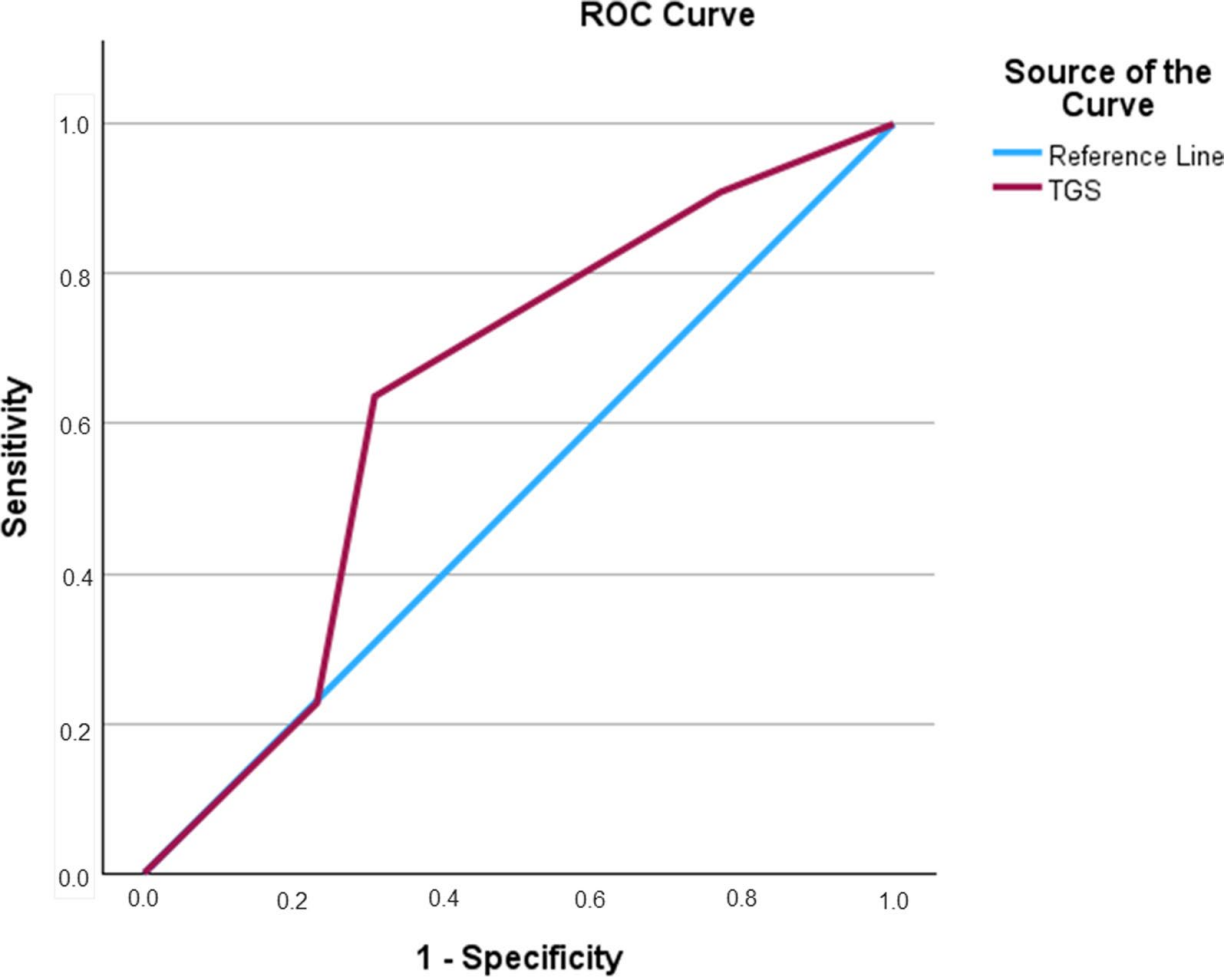
Receiver operating characteristic curve (ROC) summarizing the ability of the total genotype score (TGS) to distinguish the severity of muscle injuries

ROC analysis showed significant discriminatory accuracy of TGS in the identification of injuries in professional football players (AUC = 0.873; 95%CI 0.788–0.958; *p* < 0.0001) (sensitivity = 0.857, specificity = 0.241) (Fig. 2). The corresponding TGS value at this point was 56.2 a.u. Football players with TGS lower than 56.2 a.u. (*n* = 37) showed a mean incidence of muscle injuries of 2.28 (± 2.80), while football players with TGS higher than 56.2 a.u. (*n* = 27) showed a mean incidence of muscle injuries of 0.12 (± 0.28). In detail, subjects with a TGS lower than 56.2 a.u. had an odds ratio (OR) of 3.5 (95%CI 1.8–6.8; *p* < 0.001) to suffer an injury when compared to those players with a TGS above this value. The TGS was a statistically significant predictor of the incidence of muscle injuries (*R*^2^ = 0.29, *p* < 0.001). No significant association was observed between TGS and the severity of muscle injuries (*R*^2^ = 0.02, *p* = 0.349). ROC analysis did not show significant discriminatory accuracy of TGS in the identification of the severity of muscle injuries (AUC = 0.636; 95%CI 0.433 to 0.840; *p* = 0.189) (sensitivity = 0.636, specificity = 0.308) (Fig. 3).

## Discussion

The main finding of this study suggests that the combination of *ACE* I/D (rs4341), *ACTN3* c.1729C > T (rs1815739), *COL5A1* C > T (rs2722) and *MCT1* c.1470A > T (rs1049434) polymorphisms has influenced injury incidence in top-level football players. The results suggest a strong association (*P* < 0.001) between TGS scores and muscle injury incidence, with the *ACE* DD genotype, the *ACTN3* CC genotype, the *COL5A1* CC genotype, and the *MCT1* TT genotype that showed a protective effect against the incidence of muscle injury. Moreover, the combination of the four polymorphisms included in the model shows that the likelihood of sustaining an injury in professional football players was higher in the TGS > 56.2 a.u. group than in their < 56.2 a.u. counterparts; however, the severity of injury does not seem to be influenced by the polymorphisms included in the model (*p* > 0.05).

Muscle injuries, which account for over one-third of all time-loss injuries in men's professional football, have a serious impact on both players and their teams [1, 48] and many studies have shown that teams that reduce time-loss injuries often achieve greater league success [49–51]. Some athletes may be more susceptible to muscle injury than others in non-contact situations due to genetic factors, which could also explain why some athletes are at more risk than others.

Similar to the phenotypes associated with exercise, it is anticipated that a number of genetic variations, environmental factors, and their interactions all have an impact on the complex multifactorial trait of muscle injuries [46].

Among the most studied genetic variants candidate to influence muscle injury in football are the *ACE* I/D (rs4341), *ACTN3* c.1729C > T (rs1815739), *COL5A1* C > T (rs2722), and *MCT1* c.1470A > T (rs1049434) polymorphisms.

Regarding the *ACE* I/D (rs4341) polymorphism, the present study supports previous findings that showed the protective effect of the D-allele against muscle injuries [7, 43] and muscle damage. More in detail, de Almeida et al. [43] recently showed that Brazilian professional football players with the *ACE* II genotype had almost twofold the number of injuries per season compared to those with the ID + DD genotypes (*p* = 0.03). In support of these findings, the study of Yamin et al. [21] observed different levels of circulatory CK between the different *ACE* genotypes after eccentric exercise, suggesting that the D-allele could have a protective effect against muscle damage after training. Moreover, Sierra et al. [52] reported an association between *ACE*-related polymorphisms and inflammation and muscle damage in Brazilian male runners after a marathon race, suggesting that the *ACE* DD genotype decreases the susceptibility to inflammation and muscle damage after exercise. The low inflammatory response and muscle damage in D allele carriers may explain the association between the *ACE* I/D (rs4341) polymorphism and muscle injury in elite football players in our previous study [7] and the present study.

Regarding the *ACTN3* c.1729C > T (rs1815739) polymorphism, our results support previous findings that showed the protective effect of the R-allele against muscle injuries [34, 43] and muscle damage [35]. Pimenta et al. [35] found that CK (4-h post), alpha-actin (post and 2-h post), and cortisol (post) levels were higher among football players with the TT genotype than CT and CC athletes following eccentric training. According to a recent systematic review, athletes with the *ACTN3* TT genotype may be more likely to sustain certain indirect injuries, such as muscle damage, ankle sprains, and higher levels of exercise-induced muscle damage, which could hurt injury frequency and severity in comparison with CT and CC genotypes [53].

The expression of alpha-actinin-3 has been suggested to be a protective mechanism against the development of lesions, through an increase in type IIa muscle fiber stiffness [54], and it could be the reason for the increased susceptibility to muscle damage in the *ACTN3* TT genotype.

In support of this hypothesis, there is the up-regulation of several Z-line proteins in α-actinin-3 deficient muscle [55], which disrupts the normal protein complexes at the Z-line, altering its structural properties and suggesting a higher susceptibility to skeletal damage induced by the muscle contraction [56].

Regarding the *COL5A1* C > T (rs2722) polymorphism, the present study showed a lower incidence of injuries in CC carriers than CT carriers and a tendency toward a higher frequency of the “worst” genotype (TT) in the injured group compared to the non-injured group (*p* = 0.079). The *COL5A1* rs12722-TT genotype has recently been linked to a greater estimated number of total muscle-related injuries per game than the CC and CT genotypes (*p* = 0.028), according to research on 44 professional Australian football players [57], and previous findings showed the protective effect of the C-allele against severe muscle injuries [11]. Muscle fiber maintenance and repair depend heavily on the extracellular matrix of skeletal muscles, which is composed of the collagen type V 1 chain protein produced by the *COL5A1* gene. The *COL5A1* C > T (rs2722) polymorphism affects the stability of mRNA and the formation of type V collagen and it can also affect flexibility and range of motion (ROM) via passive muscular stiffness [58], which in turn affects the susceptibility to muscle damage [58]. More type V collagen 1 chain may be produced from the T allele than the C allele because the T allele has improved mRNA stability in comparison with the C allele [58]. According to Collins and Posthumus, an increase in type V collagen 1 chain modifies the structure of collagen fibrils, changing the mechanical characteristics of connective tissues [58].

Regarding the *MCT1* c.1470A > T (rs1049434) polymorphism, the present study supports our previous findings on professional football players from different levels that showed the protective effect of the T-allele (490-Asp) against muscle injuries [13]. *SLC16A1*, commonly referred to as *MCT1*, encodes the MCT1 transporter in humans. Merezhinskaya et al. [38] showed, for the first time, that two patients with the minor T-allele (490-Asp) had a decrease in the rate of erythrocyte lactate transport of between 35 and 40%. Later on, Cupeiro et al. [59] found that *MCT1* T allele carriers accumulated more lactate in the blood after high-intensity circuit weight training than individuals with an AA (Glu/Glu) genotype. A couple of years later, the *MCT1* c.1470A > T (rs1049434) polymorphism has been associated with sprint/power athletic status [60], body composition [40], football player status [61], Polish climbers [41], and blood lactate concentration after exercise in Japanese wrestlers [62]. More recently, research on endurance athletes revealed that those with the main A allele (Glu-490) had higher VO_2 max_ and less lactate buildup in their blood after vigorous exercise than individuals with the TT genotype (Asp/Asp) did [63]. As already discussed in our previous finding on *MCT1* c.1470A > T (rs1049434) polymorphism and muscle injury [13], we could speculate that the highest incidence of muscle injuries observed in football players with the AA (Glu/Glu) genotype could be sought in the elevated lactate transport rate from arterial blood into the muscle fibers for its oxidation, as highlighted in previous study [64]. The elevated lactate transport rate could result in an acidic intracellular environment created by muscle activity, with consequent degeneration of muscle and release of myoglobin and creatine kinase [66–68]. This factor might compromise extreme performance in healthy individuals and, considering that muscle fatigue has been shown to predispose to injury [68], lactate transporter variations in skeletal muscle might provide an explanation for muscle injuries due to the higher intramuscular lactate concentration.

Finally, the combined effect of the four polymorphisms analyzed in the present study and implicated in muscular performance and muscle flexibility significantly affected the probability of a football player getting injured. In detail, the sum of the “protective” genotypes was significantly lower in the non-injured than injured football players, while the TGS was a predictor of muscle injury incidence.

The importance of analyzing the combined influence of different candidate gene polymorphisms on complex phenotype traits, such as muscle injuries and/or athletic performance, has recently been shown [10, 42]. Our results agree with the recent finding of Maestro et al. [10], who analyzed the influence of some polymorphisms (*AMPD1, ACE, ACTN3, CKM, *and *MLCK*) on muscle injuries in soccer. The authors showed significantly higher TGS value in non-injured soccer players compared to injured soccer players (57.1 a.u. vs 51.7 a.u., respectively, *p* = 0.034). Moreover, they discovered a TGS cut-off point of 45.8 a.u. to discriminate non-injured from injured soccer players, showing that players with a TGS beyond this cut-off had an odds ratio of 1.9 (95%CI 1.1–2.9; *p* = 0.022) to suffer an injury when compared with players with lower TGS.

Varillas-Delgrado et al. [42] found that the combined influence of some selected polymorphisms (*ACE, ACTN3, AMPD1, CKM*, and *MLCK*) showed a favorable odds ratio of being a professional athlete against a non-athlete in muscle injuries (OR 2.7; 95% CI 1.7–4.1; *p* < 0.001). The same authors [69] recently showed that TGS analysis, combining influence of *AMPD1* (rs17602729), *ACE* (rs4646994), *ACTN3* (rs1815739), *CKM* (rs8111989) and *MLCK* (rs2849757 and rs2700352) polymorphisms, appears to correlate with elite endurance athletes at higher risk for injury.

The results of this pilot study, according to the previous finding [10, 42, 69], highlight that muscle injuries are a very complex phenotype trait influenced by multiple gene variants, suggesting the need to develop a genetic risk score profile for muscle injuries in professional football players. In future, this would allow the coaches and the medical staff of the football teams to adapt the training protocols according to the genetic risk profile of the football players to avoid overloading the athletes most at risk of developing muscle injuries.

The long-term objective of this research is to develop a polygenic risk profile that includes an increasing number of genes affecting the inter-individual variability in muscle injury incidence and severity among football players. However, it is also necessary to highlight that the major limitations of the present study are represented by the small sample size utilized for the analyses, which means that future studies are needed to replicate the results in different football player cohorts. Moreover, new SNPs implicated in muscle injuries in non-Italians professional football players have been discovered, such as *AMPD1* [10] and *CKM* [42], that have not been included in our model and can also explain individual variations, together with other numerous genetic variants implicated in the susceptibility to develop muscle injuries in football. Last but not least, our conclusions are referred only to the male football players, highlighting the need to replicate our findings also in the female football player’s cohort.

On the other hand, the strengths of the present study are represented by the extreme homogeneity of the sample examined, the methodology to collect and classify the injuries, and the longitudinal injury data collection, which allow us to state that the results obtained are representative of the reference sample. Compared to our previous studies, the sample of the present work was selected including in the analyses only football players of the first team who had participated in the First League Italian Championship (Serie A). Because the incidence of injuries can be influenced not only by the training exposure but also by the type of training, the pitch, the age, and the level of the athletes [70], we consider it a very important aspect in reducing the number of confounding variables even to the detriment of the decrease in the sample size.

Finally, our preliminary data suggest that a suitable polygenic profile might help to reduce the likelihood of developing muscle injury during football training and matches and this study contributes to the literature on the subject that aims to identify the genetic profile of players at high risk of developing muscle injuries. Future practical applications of this type of research will be to create individualized training programs, modulating the main training load parameters (Volume, Intensity, Specificity, Recovery, and Density) based on a football player’s genetic characteristics, preserving in that way the athlete's health with high genetic risk to develop muscle injuries.

## Conclusion

Adapting the training load parameters to the athletes’ genetic profile represents today the new frontier of the methodology of training. In this pilot research, we have performed a preliminary, small-scale study that will be in future replicated increasing sample size and adding more genetic markers associated with muscle injuries. Further studies using different cohorts of professional football players all around the world are needed to replicate this finding and to include in the TGS more potential genetic variant candidates to influence muscle injuries in football.

## Data Availability

All data generated or analyzed during this study are included in this published article.

## Abbreviations

ACL: Anterior cruciate ligament
*ACE*: Angiotensin-converting enzyme
*ACTN3*: Alpha-actinin-3
AIC: Akaike Information Criterion
*COL5A1*: Collagen Type V Alpha 1 Chain
CK: Creatine kinase
DNA: Deoxyribonucleic acid
EIMD: Exercise-induced muscle damage
GS: Genotype score
*MCT1*: Monocarboxylate transporter 1
PCR: Polymerase chain reaction
SNP: Single nucleotide polymorphism
TGS: Total genotype score
UEFA: Union of European Football Associations
